# Availability and Accessibility of Student-Specific Weight Loss Programs and Other Risk Prevention Health Services on College Campuses

**DOI:** 10.2196/publichealth.5166

**Published:** 2016-06-08

**Authors:** Sarah Lynch, Sharon Hayes, Melissa Napolitano, Katrina Hufnagel

**Affiliations:** ^1^ Department of Psychology University of Colorado Denver Denver, CO United States; ^2^ Keiser University Department of Psychology Port St. Lucie, FL United States; ^3^ The George Washington University Department of Prevention and Community Health; Department of Exercise Science Milken Institute of Public Health Washington, DC United States; ^4^ The George Washington University Department of Prevention and Community Health Milken Institute of Public Health Washington, DC United States

**Keywords:** weight loss, student health services, students, universities

## Abstract

**Background:**

More than one third of college students who are overweight or obese are in need of weight loss programs tailored to college students. However, the availability and accessibility of these programs is unknown.

**Objective:**

The aim of this study is to examine the availability and ease of access to weight loss programs for students at 10 universities with the largest undergraduate enrollment.

**Methods:**

The 10 public universities with the largest student bodies with a mean (SD) undergraduate enrollment of 41,122 (7657) students were examined. The websites of the universities were assessed to determine the availability of weight loss programs. Services for high-risk health needs common to university campuses (ie, alcohol and other drugs, victim services, sexual health, and eating disorders) were searched.

**Results:**

Of the universities searched, 3 (30%, 3/10) offered weight loss programming, however, none met the predetermined criteria. Comparatively, all schools (100%, 10/10) offered no-cost and continual enrollment programming for the other high-risk health needs.

**Conclusions:**

There are limited weight loss services available to undergraduate students compared with other university services. Collaboration between existing college health service providers is suggested for the delivery of appropriate programming for overweight and obese undergraduates wanting to lose weight.

## Introduction

College campuses provide a foundation for the development of lifelong behaviors for over 70% of young adults between the ages of 18 and 24 who enroll in college [1]. During the undergraduate years, young adults must adjust to multiple personal, social, and academic demands while trying to establish or maintain healthy lifestyle behaviors [2]. This adjustment can be challenging particularly when it comes to establishing healthy eating and exercise patterns [2]. Services offered on university campuses are a convenient resource for undergraduate students looking for assistance with the college adjustment. Undergraduate students’ need for university-based health services has increased over the past 5 years, as evidenced in a 2013 survey of college counselors [3].

Services for high health risk needs, like alcohol and other substance use, eating disorders, sexual health services, and victim services are generally recognized as critical to ensure the health of undergraduate students and other university members. However, the prevalence of the high health risk needs requiring services on college campuses varies dramatically. For example, the prevalence of undergraduate students with alcohol use disorders ranges between 20.4% [4] to 31% [5]. According to the Centers for Disease Control and Prevention, 1 in 4 sexually active young adults between the ages of 19 to 24 years have a sexually transmitted disease [6]. Rates of sexual assault in undergraduates range from 1.9% to 7% in college populations [7]. With regards to eating disorders, 1.2% of undergraduate females report being diagnosed and/or treated for anorexia [7], 1% for bulimia [7], and 8.4% for binge eating disorder [8]. An additional 9% of young women meet Diagnostic and Statistical Manual of Mental Disorders, Fifth Edition (DSM-V) criteria for specified or unspecified feeding or eating disorder [9,10]. Comparatively, approximately 35% percent of college students are overweight or obese [7].

As with the services for high-risk needs described above, colleges have the unique opportunity to address weight and weight-related topics to further ensure the health of their student body. Average weight gain from college matriculation to graduation ranges from 3.5 pounds [11] to 9.5 pounds [12]. Of note, upon college matriculation, 22% of college students are already overweight, and 12% are obese [7]. With one-third of undergraduate students already at risk for negative health and psychosocial outcomes associated with obesity [13,14], relatively small weight gains can increase an individual’s risk of having a body mass index (BMI) in the overweight or obese category [15]. These small gains may increase an individual’s risk for type 2 diabetes, cardiovascular disease, and depression [16-19].

Despite this public health concern, there remains a need on campuses for weight management services tailored to undergraduates [20,21]. Behavioral health services for the treatment of obesity have lagged behind other on-campus high-risk health behavior services, including drug and alcohol use, risky sexual behavior, eating disorders, and victim services [22]. A 2013 survey of university-based registered dietitians found that over half of participating university dietitians felt it was important for their respective universities to offer structured weight loss programming for students [23]. An integrative approach to the treatment of overweight and obesity could provide an opportunity for counseling centers and health centers to collaborate by promoting the application of evidenced-based practices and access to mental health resources that individuals may need while losing weight. However, it is unclear what services are currently available for overweight and obese undergraduate students wanting to lose weight. No systematic assessment of weight loss programs specific to college students and offered on university campuses could be located in the literature.

Research suggests that weight gain [24] and access to weight-related services [25] are concerns for undergraduate students. A recent study reviewing causes of stress referenced in undergraduate student Facebook pages found weight gain was referenced in 10% of posts [24]. A qualitative study of undergraduate students found that students who felt they had greater access to fitness facilities and healthy eating options were more likely to use those services [25]. Despite this evidence, it remains unclear as to what weight loss services are available and accessible to overweight and obese undergraduate students wanting to lose weight.

This study examines the availability and ease of access of weight loss programs for undergraduate students compared to existing services for other high-risks health needs (ie, alcohol and other drugs, eating disorders, victim services, and sexual health).

## Methods

### Search Methodology

The 10 public universities with the largest undergraduate student bodies, with a mean (SD) undergraduate enrollment of 41,122 (7657.4) students, were identified using the 2013 US News and World Report College Guide [26]. Because undergraduate students may be more comfortable in programming specifically for students of their same age and demographic, particularly ones that do not include graduate students or faculty who may be their professors or advisors, we selected to focus on weight loss programs tailored specifically to undergraduate students. Similarly, health programing for other high-risk health services are traditionally tailored exclusively to the needs of undergraduate students; as such, we decided to use this same metric when examining weight loss programing. The Internet is ranked as the top source of gathering health-related information for college students [27]. Thus, a Web-based search was elected to search for university weight loss programs. Additionally, college students report trusting health information originating from their respective universities and university health centers [7,27]. For this reason, only websites with edu links were reviewed.

Structured searches of university pages using standardized search terms were conducted over a period of 4 months (July 2013 to October 2013). Search terms were typed into the university’s main page by 3 independent coders (2 undergraduate and 1 graduate research assistants). Phone calls were placed to university health, counseling, and wellness centers to confirm the Web search findings.

Search terms were initially selected through a brief survey of undergraduate students that asked students to identify what term they would use to search for weight loss services on their campus. The suggested search terms were reviewed by 2 clinical psychologists and a registered dietitian with experience in the development and delivery of undergraduate weight loss programming, as well as by 2 undergraduate and 2 graduate research assistants. A total of 15 key terms were used to search for student-specific weight loss programs using the search box found on the home page of the respective universities: diet program, weight loss program, losing weight, fitness program, nutrition counseling, nutritionist, dietician, dietitian, exercise facility, weight watchers, student weight loss, student diet program, student weight loss program, weight management, and student weight management *.*

Search terms for traditional high-risk health services offered on university campuses (ie, alcohol and other drugs, victim services, sexual health, eating disorders) were included to compare the availability of these services to student weight loss services. These terms were also typed in the search box found on the home page of the respective universities.

Search terms were additionally typed into the search box on the student health services page and university counseling pages to ensure no programs were not found due to website-derived search algorithms unknown to the research team.

### Establishment of Inter-Rater Reliability

The aforementioned clinical psychologists established criteria prior to the webpage reviews. In order to be coded as a weight loss program, the program must have been undergraduate student-specific, offered on campus, no additional cost, and delivered by university-funded treatment providers. Results were also coded as “related weight loss services” (missing one or more of the weight loss program criterion, fee-based weight loss program, nutrition counseling, personal training, research on college student weight loss or community-based weight loss program), or no weight loss program (group fitness programs, academic research with a focus outside the scope of college student weight loss, nutrition and physical activity handouts).

Two undergraduate and one graduate research assistant piloted the search terms on 4 large (undergraduate student enrollment greater than 30,000 students) test universities, which were randomly selected from a list of universities from the US News and World Report [26]. These 4 schools were not included in the final university sample pool. Terms were typed into the university search engine found on the university’s home page. Research assistants took a screen shot of the results and a screen shot of the first page of each link clicked on, in order to capture the content of the website at the time reviewed. After completion of coding, screen shots and coding of the content were reviewed and discussed between the coders. Coding decisions were confirmed under the direction of 2 clinical psychologists who have experience in the development and delivery of college weight loss treatments.

## Results

### Weight Loss Programs

Although the predetermined criteria were not met, the following 3 universities offered some weight loss programming: (1) University of Minnesota offered a weight loss program for their undergraduate students; however, the 12-week program for undergraduates cost US $250, (2) Florida International University offered an 8-week program for undergraduate students inspired by the Biggest Looser for US $80, and (3) University of Texas-Austin offered a 12-week program exclusively to graduate students for US $60. Phone calls placed to university health, counseling, and wellness centers verified the results. The aforementioned programs had discrete enrollment periods. Two other universities offered short-term programs, but there was no confirmed plan to continue those regularly beyond the planned dates of the one program. For the purpose of this paper those programs were not included.

### Accessibility of Weight Loss Programs

The results of our findings are shown in Table 1. Coders spent a mean (SD) of 102.2 (21.0) minutes searching for weight loss programs using the15 search terms, and a mean (SD) of 8.2 (6.8) minutes per search term. Comparatively, coders spent a mean (SD) of 20.5 (2.9) minutes searching for 4 traditional university services and 5.1 (0.7) minutes per search term. Although the predetermined criteria were not meet, 3 universities offered student weight loss programs. Comparatively, all of the universities searched (100%, 10/10) offered free, university-sponsored, student-specific services for sexual health and victim services. We found that 90% (9/10) of the universities offered free, university-sponsored student-specific services for alcohol and other drug treatments; one school required a fee for assessment and treatment. In addition, 90% (9/10) universities offered free, university-sponsored student-specific services for eating disorders; one school provided screening and referred out to the community for treatment.

## Discussion

### Principal Findings

Three schools offered weight loss programming, however, none met the predetermined criteria. Comparatively, all schools offered no-cost and continual enrollment programming for the other high-risk health needs. Given the current prevalence of overweight and obesity among college students and the demonstrated weight gain during college, there is a need for obesity-related services on campuses. Universities have competing wellness priorities for their students, and they may designate resources for those behavior and health risks that have more immediate negative consequences such as suicide or alcohol-related death or injury [28-30]. Obesity treatment may be a lower priority as the consequences may not manifest until long after students leave the university. However, our findings indicate that some colleges are beginning to offer services specific to the treatment of obesity in their college populations.

The focus of weight management on college campuses may reflect changes in health services mandated by the Affordable Care Act (ACA). Students are now able to remain on their parents’ insurance until the age of 26 years old [31]. The extended insurance coverage is coupled with an increased focus on obesity prevention and weight loss under the ACA, which requires insurance providers to provide regular BMI screening, nutrition counseling, and other weight management services for no or little cost to the insured [32]. The 35% prevalence rate of overweight and obesity in undergraduate students [7] is comparable to or exceeds the prevalence rates of high-risk behaviors that have regularly provided no-cost services (10% for eating disorder [7-10], 20-30% for alcohol and other drug use [4,5], 25% of sexually transmitted disease [6], and 1-7% for sexual assault [7]). Given the shift in focus that we have seen nationally related to weight management due to the ACA, colleges have the opportunity to mirror these new initiatives.

**Table 1:** The largest public universities and the health services offered (N=10).

University	Number of students^a^	Weight loss program	Victim services	Sexual health	Alcohol and other drugs	Eating disorder treatment
University of Central Florida	51,269	0^b^	X^c^	X	X	R^d^ (only screening offered)
Texas A&M	44,315	0	X	X	X	X
Ohio State	44,201	0	X	X	X	X
Penn State	40,085	0	X	X	R (fee)	X
University of Texas at Austin	39,979	R (fee; only for graduate students)	X	X	X	X
Florida International University	39,045	R (fee)	X	X	X	X
Arizona State University	38,735	0	X	X	X	X
Michigan State	37,988	0	X	X	X	X
University of Minnesota	34,449	R (fee)	X	X	X	X
University of Florida	33,168	0	X	X	X	X

^a^Academic year 2013-2014.

^b^0 is no program.

^c^X is a program that meets established criteria.

^d^R is a related program that does not meet established criteria for program.

Despite these opportunities, colleges and universities are faced with a number of demands in terms of academic and health-related priorities and limited funds. Examining the barriers and facilitators on college campuses to delivering weight loss programming is an important step in understanding the broad scale implementation and acceptability of these programs. One on-going project, funded by the National Institutes of Health (DK 100916), is examining the efficacy of using social media and text messaging for delivering weight loss programming to college students on 2 different campuses [33]. In addition to examining weight loss and metabolic outcomes (ie, glucose, blood pressure, lipids), one primary aim of this project is to evaluate the implementation feasibility and sustainability infrastructure on college campuses for delivering weight loss programs tailored for students. This evaluation will include a cost effectiveness assessment as well as interviews with key administrative and student leaders on campus to provide data on both the facilitators and challenges to offering tailored weight management programs.

A 2011 analysis of the American College Health Association-National College Health Assessment (ACHA-NCHA) database found that the top two most trusted sources of health information were health center medical staff and health educators [34]. However, this same study found that these two sources of information were also the most under-utilized method of gathering health information [34]. While it is unclear why these on-campus resources are underutilized, it is possible that the nature of the Web search (eg, timely, cumbersome) may have been a barrier to utilization. Students relying on the Internet alone might struggle to find information about on-campus services. The cost of these programs may also reduce the likelihood that students will utilize the resources. We found weight loss programs exclusively for undergraduate students were available for a fee (US $60-$250) at 2 of the 10 universities included in this Web-based search (University of Minnesota and Florida International University).

For these and other reasons, a multidisciplinary approach to disseminating and marketing a weight loss program specific to college students is critical. Collaboration with the counseling center may be particularly important. The is concern that weight loss services on college campuses may promote disordered eating and/or eating disorders in a vulnerable college population [35]. Dieting is prevalent on college campuses: over 60% of college females and 36% of college males report they are trying to lose weight, and 48% of college females and 28% of college males reported dieting to lose weight [7]. Overweight and obese young adults who desire to lose weight are just as likely to develop an eating disorder as the general population [36], yet eating disorder treatment for this population is often over looked [23,37].

There is literature suggesting that there may be a common underpinning for both excessive weight gain and eating disorders. These shared experiences and characteristics are dieting, media use, body image dissatisfaction, and weight-related teasing [38,39]. Thus, it is not weight loss per se, but a combination of maladaptive behaviors and factors that may put an overweight or obese individual at risk for an eating disorder. College women, in particular, may use a combination of healthy and unhealthy weight loss practices [40]. Thus, a multimodal approach of social, environmental, and individual strategies coupled with media literacy and advocacy is needed for overweight and obese students wanting to lose weight [23,41]. It is critical that university health providers delivering weight loss programs for students be aware of the possibility of underlying eating disorders that may complicate the students’ efforts to lose weight so that the appropriate treatment can be delivered to students [23,37]. The key is to provide education and evidence-based programs to teach safe, effective, and healthy ways of losing weight.

While many university health officials and administrators recognize the need for an environment that promotes healthy weight loss practices and relevant programming, there are a number of challenges faced by these key leaders on campus. There are competing priorities for resources, time, as well as other behavioral and health needs with more immediate consequences [28,29,42]. Unfortunately, outsourcing weight loss programming is likely not the solution to implementing weight loss programs for undergraduate students as traditional weight loss programs for adults. Young adults who attend weight loss programs tailored to adult populations attend fewer sessions and lose less weight than adults attending weight loss programs [43]. Many universities likely already have necessary components that would allow them to offer a systematic program to prevent and treat obesity. The key is to integrate these available services and personnel to provide education and evidence-based programs that are attuned to the potential for disordered eating behaviors in this population of overweight and obese students wanting to lose weight [40]. Programming that promotes a campus environment of positive body image and self-esteem may help overweight and obese and normal weight students who want to lose weight. For example, The Body Positive is a body image and health curriculum for high school students that promote physical and mental health through workshops, videos, and campus events. These types of programs could provide an interim solution prior to the launching of sustained weight loss program offerings.

### Limitations

This study provides only a snapshot of the availability and ease of access to weight loss services on college campuses. Only the websites of the ten largest public universities were reviewed, limiting the search and possibly ignoring student weight loss programs available at smaller and/or private universities. Universities often update their webpages so that recent content is pushed to the top of searches, so it is possible that the results presented here were influenced by search mechanisms unknown to the study authors. Lastly and importantly, this study was not able to determine the extent of the utilization of available weight loss services by university students.

### Conclusions

This study examined the avaliability and ease of access to weight loss programs for college students. The results highlight that there are limited weight loss services available and accessible to college students when compared to other university services, such as alcohol and other drugs, eating disorders, sexual health, and victim services.

Advocacy from student groups and collegiate administrators is called for to provide treatment and reduce stigma regarding accessing resources specific to weight loss for overweight and obese students wanting to lose weight.

## Abbreviations

ACA: Affordable Care Act
BMI: body mass index
